# The Needs, Use and Expectations of People Bereaved by Suicide Regarding Online Resources: An Online Survey

**DOI:** 10.3390/ijerph191912156

**Published:** 2022-09-26

**Authors:** Edouard Leaune, Héloïse Rouzé, Laurène Lestienne, Kushtrim Bislimi, Benoit Chalancon, Margot Morgiève, Pierre Grandgenèvre, Guillaume Vaiva, Nathalie Laplace, Emmanuel Poulet, Julie Haesebaert

**Affiliations:** 1 Centre Hospitalier Le Vinatier, 69678 Bron, France; 2 INSERM, U1028, CNRS, UMR5292, Lyon Neuroscience Research Center, Psychiatric Disorders: From Resistance to Response—PSYR2 Team, 69000 Lyon, France; 3 Groupement d’Etude et de Prévention du Suicide, 29200 Brest, France; 4 Research on Healthcare Performance RESHAPE, INSERM U1290, Université Claude Bernard Lyon 1, 69100 Villeurbanne, France; 5 Service Recherche et Epidémiologie Cliniques, Pôle Santé Publique, Hospices Civils de Lyon, 69003 Lyon, France; 6 Cermes3, Université Paris Cité, CNRS, Inserm, 75006 Paris, France; 7 U1172-LilNCog-Lille Neuroscience & Cognition, University of Lille, Inserm, CHU Lille, 59000 Lille, France; 8 Centre National de Ressources & Résilience Pour Les psychotraumatismes (Cn2r Lille Paris), 59000 Lille, France; 9 Interlude Santé, 69530 Brignais, France; 10 Department of Emergency Psychiatry, Hospital Edouard Herriot, Hospices Civils de Lyon, 69003 Lyon, France

**Keywords:** suicide, bereavement, online resource, survey

## Abstract

Background: Online resources constitute a new and effective way to obtain support or information during bereavement processes. However, little is known about the needs, use and expectations of people bereaved by suicide regarding online resources. Method: The objective of our national cross-sectional online survey was to collect the use, needs and expectations of people bereaved by suicide regarding online resources. The data were collected from July to October 2021 through a 26-item online questionnaire hosted on the website LimeSurvey. Results: A total of 401 respondents fully completed the questionnaire. Their mean age was 45.7. The majority of participants were women bereaved by the suicide of their child or partner. Half of the participants were bereaved for less than 3 years and benefited from counselling during their bereavement process. Three-quarters of the participants used the Internet for their bereavement process, mainly to obtain information on suicide bereavement and suicide prevention and to access testimonies of other people bereaved by suicide. Three-quarters of the participants found that available online resources for people bereaved by suicide are insufficient and expected a dedicated web platform to be developed. Finding information on suicide bereavement and on suicide prevention, discussing with a mental health professional and accessing testimonies of other people bereaved by suicide were expected by a majority of the participants regarding the future platform. Receiving counselling and being bereaved by the death of a child were the most important factors in explaining patterns of use and expectations regarding online resources. Discussion: Our results offer precise insights into the needs, use and expectations of people bereaved by suicide regarding online resources. The development of web platforms offering access to reliable information on suicide bereavement and on suicide prevention to peers bereaved by suicide and help to seek counselling are urgently needed.

## 1. Introduction

Suicide bereavement is known to be highly prevalent in the general population, as more than one person in five is exposed to the suicide of a relative during their lifetime [1]. Suicide bereavement is also known to be associated with high levels of distress [2,3], including mental health problems such as suicidal ideation and behaviours, complicated grief, acute and post-traumatic stress disorders, mood and anxiety disorders [4] or substance use [5]. According to recent epidemiological studies, the prevalence of complicated grief (from 25% to 43%) and suicidal ideation (from 14% to 49%) are particularly high [4]. Specific grief processes have been identified in suicide bereavement, including guilt and shame, stigma, loss of life meaning and high intensity of psychological turmoil [2]. The deleterious psychosocial impact of suicide bereavement through high levels of perceived stigma is associated with social withdrawal [6] and dropping out of education and work [7]. Physical adverse outcomes, especially pain, cardiovascular disease, hypertension, diabetes and chronic obstructive pulmonary disease, are also frequent consequences of grief in people bereaved by suicide, which could lead to short- and long-term disability [8]. However, people bereaved by suicide can experience post-traumatic growth, defined as a significant positive change experienced by an individual following stressful or challenging life events in their life in the months and years following the loss of their relative [9], especially when receiving effective social support [10]. In a recent meta-analysis, help-seeking, perceived social support, time since loss and adaptive coping strategies support were found to be significantly associated with post-traumatic growth in people bereaved by suicide [10].

Online resources constitute a new and effective way to obtain support or information during bereavement processes. For example, according to a recent meta-analysis, more than half of all bereaved people use digital resources for their grief work [11]. These online resources are diverse and can include informative websites about grief and loss, memorial websites, online support groups and online therapy and counselling, of which the most commonly used are online support groups, social media and memorial websites [12]. According to a recent systematic review [13], online resources can enhance early access to help and support for people bereaved by suicide, for whom the need for early and pro-active postvention interventions is reported [14]. Several studies reported that women, parents bereaved by the loss of a child and people bereaved for less than 5 years receiving counselling, having lower income, experiencing more social stigma and feeling more isolated are more prone to use online resources for their bereavement process [13]. Regarding the age of users, most of them are reported to be middle-aged, and low participation of adolescents and young adults is reported in the literature [13]. Interestingly, the literature also indicates that online resources may be of particular interest to reaching people who might otherwise not be able to access the support they need because of personal and structural barriers [13]. Despite a shortage of evidence on the effectiveness of online resources, most studies report perceived benefits [13], such as the possibility of using the resources around the clock and discussing grief-related topics without being judged. While the important need for support for people bereaved by suicide is often not fulfilled, online resources are a much-needed addition to available, mostly in-person, resources. However, more research is needed in order to build evidence-based online resources for people bereaved by suicide.

The objective of our study was to collect the use and expectations of people bereaved by suicide regarding online resources and social media. We aimed to assess how people bereaved by suicide use the Internet and social media during their bereavement process. We also aimed to assess their expectations regarding the development of a new online resource dedicated to suicide bereavement.

## 2. Methods

### 2.1. Data Collection

This survey is part of the ESPOIR_2_S study, a mixed-method collaborative and participatory user-centred study. The protocol of the study, relying on the active participation of users, has been exhaustively described elsewhere [13]. ESPOIR_2_S seeks to build resources from the perspectives and needs of both people bereaved by suicide and professionals or volunteers working in the field of postvention through the iterative process of the *Information System Research (ISR) framework*. The ISR framework employs various design processes to build a product or design an artefact such as a mental health online resource [15,16]. We describe in this article the results of the first quantitative stage of the study, namely the *relevance cycle,* which aimed to collect the use, needs and expectations of people bereaved by suicide. We used the *Checklist for Reporting Results of Internet E-Survey* (CHERRIES) to guide reporting of results [17]. We conducted a national cross-sectional online survey in France from July to October 2021. The data were collected through a 26-item online questionnaire that was built by our pluriprofessional research team for the study according to a recent systematic review of online resources for suicide bereavement [13] and was hosted on the website LimeSurvey. This newly generated questionnaire collected six sociodemographic and loss-related characteristics (age, gender, relation to the deceased, time since loss, access to counselling and type of counselling) and evaluated four dimensions: (1) three items on the use of the Internet and social media in daily life; (2) one item on perceived needs regarding suicide bereavement (social support, professional counselling, peer support, meaning-making); (3) nine items on the use of online resources associated with suicide loss (frequency, type(s) of resources, reasons for using online resources, satisfaction with the resources); and (4) seven items on the expectations regarding the development of an online resource for people bereaved by suicide (types of resource, technologies, typology of use) and personal propositions regarding the development of an online resource for people bereaved by suicide (type of resource, technologies, usability). The full questionnaire is available in the Supplementary Materials.

### 2.2. Participants and Recruitment

All persons bereaved by suicide were eligible to participate in the study. As the questionnaire was in French, being able to read and write in French was the only condition for participation. The link to the questionnaire was proposed to putative respondents through social media (Twitter, Facebook, Instagram) and professional and associative mailing lists between July 2021 and October 2021. A purposive snowballing recruitment process was, thus, used to build a convenience sample with the aim of covering a broad range of sociodemographic profiles. No incentive was proposed for questionnaire completion.

### 2.3. Sample Size

A purposive snowballing was, thus, used to build a convenience sample. According to the measure of sufficient sample in online surveys reported in a recent study [18], we estimated that the participation of 385 respondents would permit us to estimate a proportion with a 95% confidence interval and a 5% error margin. Due to the method of dissemination of the online survey by convenience sampling, we were not able to define the size of our source population, so we chose the highest estimate of 385 as the sample size for the online survey, as defined by Adam (2018) [18].

### 2.4. Data Analysis

All data were collected anonymously. We analysed complete cases. Qualitative variables were described by numbers and frequencies and compared using the Chi^2^ test or Fisher’s test when appropriate. Quantitative variables were expressed as mean ± standard deviation and compared using the Student’s *t*-test or the Wilcoxon test in the case of non-normal distributions. We used univariate and multivariate logistic regression models to analyse the association between reported use of the Internet or the expectations regarding online resources and several putative associated factors. We chose to focus the analyses on the use of the Internet, as it was reported as the online resource most used in the aftermath of the death and that an Internet web platform was expected for the future online resource. For the univariate and multivariate analyses, four dimensions of use or expectations were built, namely: (1) reaching peers, (2) obtaining information, (3) seeking counselling and (4) memorialising. Five variables were identified as putative explaining factors of use and expectations, namely: (1) age, (2) gender, (3) relationship with the deceased, (4) duration of bereavement and (5) receiving counselling. Age was dichotomised into two categories depending on the median age of the sample (more or less than 46 years old). Duration of bereavement was dichotomised into two categories (more or less than 3 years). All tests were two-tailed, and a *p*-value of <0.05 was considered significant. All analyses were performed using the SAS 9.2 software (SAS Institute Inc., Cary, NC, USA).

### 2.5. Funding Sources and Ethical Approval

The ESPOIR_2_S study is funded by the *Scientific Research Committee from the Centre Hospitalier le Vinatier* (funding number CSRN05) and by the *National Institute for Public Health Research* (Institut de Recherche en Santé Publique—funding number IRESP-RSP2020-230791). The study received ethical approval from the Ethical Review Board of the University Claude Bernard Lyon 1 in January 2021 (registration number 2021-01-12-04).

## 3. Results

### 3.1. Participants

The questionnaire was acceded 755 times. A total of 401 respondents fully completed the questionnaire. The majority of participants were women bereaved by the suicide of their child. A substantial proportion of our sample was bereaved by the suicide of their partner, their sibling or their parent. A slight majority of the participants were bereaved for less than 3 years and benefited from counselling during their bereavement process. The mean age was 45.7, ranging from 15 to 80. The vast majority of the participants had access to digital resources and used online resources daily. The characteristics of the participants are displayed in Table 1.

### 3.2. Needs during Suicide Bereavement Process

The need to honour the memory of their relative was the most highly rated need (extremely or very much by 66.1% of participants). Obtaining information on suicide prevention and suicide bereavement were also frequent needs, respectively reported by 58.6% and 56.3% of the participants. On the contrary, accessing online counselling was not reported as a need regarding online resources for the majority of participants (73.8%).

### 3.3. Use of Online Resources

The majority of participants frequently used the Internet and social media (Table 2). After the death of their relative, they mainly used their cell phone to reach online resources (64.8%). Nearly three-quarters of the sample (73.8%) used the Internet for their bereavement process, while nearly two-thirds (61.6%) used social media. Regarding social media, Facebook was used by the majority of participants (58.1%). WhatsApp and Instagram were also used by a substantial proportion of our sample (Table 2). Only a minority of the participants reported their use of the Internet or social media as beneficial or very beneficial for their bereavement process, respectively 29.2% for the Internet and 26.1% for social media. At the same time, few participants reported their use as not beneficial, respectively 8.6% for the Internet and 11.0% for social media.

The participants mainly used the Internet to find information on suicide bereavement (54.6%) and suicide prevention (47.9%) and to access testimonies of other people bereaved by suicide (47.6%). The use of social media was mainly associated with honouring the memory of their relative deceased by suicide (37.2%), accessing testimonies of other people bereaved by suicide (36.7%) and discussing with other people bereaved by suicide (31.2%).

When compiling the use of the Internet in four dimensions, obtaining information on suicide and suicide bereavement (71.3%) and reaching peers bereaved by suicide (57.6%) were the most frequently reported uses of online resources (Table 3).

### 3.4. Expectations Regarding Online Resources

The majority of participants reported that existing online resources for people bereaved by suicide were insufficient or largely insufficient (73.1%). A web platform (70.1%) or a specific social media (57.9%) were expected by the majority of the participants. Obtaining information on suicide bereavement (65.1%) and on suicide prevention (59.9%) were the most reported expectations regarding the online resource. Discussing with a mental health professional was also frequently expected (57.1%), but accessing online counselling was the less frequently reported expectation (27.2%). Accessing testimonies of other people bereaved by suicide was expected by a slight majority of the participants (53.4%).

When compiling expectations in four dimensions, obtaining information on suicide and suicide bereavement (77.3%) and reaching peers bereaved by suicide (65.6%) were the most frequently reported expectations (Table 3).

### 3.5. Factors Associated with Use and Expectations Regarding Online Resources

In the multivariate analysis of the use of the Internet (Table 4), parents bereaved by the suicide of their child were more prone to reach peers (*p* < 0.001), search for information (*p* = 0.007) and memorialise than other participants (*p* < 0.001). Receiving counselling was significantly associated with using online resources to reach peers (*p* = 0.004) and to obtain information (*p* < 0.001). Being bereaved for less than 3 years was significantly associated with reaching peers (*p* = 0.014) and searching for counselling (*p* = 0.012). Women (*p* = 0.049) and younger participants (*p* = 0.015) were more prone to use online resources to memorialise than other participants. People bereaved by the death of their partner were more prone to reach peers (*p* = 0.002) than others.

In the multivariate analysis of the expectations of the participants (Table 5), receiving counselling was significantly associated with using online resources to reach peers (*p* = 0.026), obtain information (*p* < 0.001) and seek counselling (*p* = 0.001). Parents bereaved by the suicide of their child were more prone to reach peers (*p* = 0.027), while participants bereaved by the suicide of their partner (*p* = 0.004) or their parent (*p* = 0.049) were less prone to use the Internet to memorialise. Younger participants were more prone to seek counselling than older ones (0.007). No significant differences were observed according to gender, duration of bereavement or for people bereaved by the death of a sibling.

## 4. Discussion

### 4.1. Summary of Results

We performed the largest online study on the needs, use and expectations of people bereaved by suicide regarding online resources. The majority of the participants were women bereaved by the suicide of their child or partner, frequently using the Internet and social media. First, the participants mainly reported the need to honour the memory of their relative and to obtain information on suicide prevention and suicide bereavement. Second, three-quarters of the participants used the Internet for their bereavement process, mainly to obtain information on suicide bereavement and suicide prevention and to access testimonies of other people bereaved by suicide. Third, three-quarters of the participants found that available online resources for people bereaved by suicide are insufficient and expected a web platform to be developed. Fourth, obtaining information on suicide bereavement and on suicide prevention and accessing peers bereaved by suicide was expected by a majority of the participants. Finally, receiving counselling and being bereaved by the suicide of a child were the most important factors in explaining patterns of use and expectations regarding online resources.

### 4.2. Discussion of Results

Consistent with previous studies, we found that a vast majority of our sample used online resources for their bereavement process. In a recent online study on 327 Swedish participants bereaved by suicide, Westerlund [19], for example, reported that 83% of the sample were active users of online support groups and/or memorial websites. The spread of digital resources in the general population offers new means to manage grief processes. Indeed, online resources can help people bereaved by suicide to overcome obstacles to receiving support via their geographic independence, 24/7 availability and anonymity, which could be important for bereaved individuals who frequently feel shame and face strong social stigma [13,20].

In our sample, honouring the memory of their relative was found to be the main need of the participants but was not reported as a frequent use or expectation regarding online resources. Memorial websites are specific online resources helping bereaved people to enhance continuing bonds with the deceased and to communicate and share the grief with others at any time and anywhere [19]. However, in the study of Westerlund [19], while online support group activity was found to be significantly associated with satisfaction regarding psychosocial health, memorial website activity showed a tendency towards a negative association. Thus, the effectiveness of memorial websites in providing support to people bereaved by suicide needs to be further assessed before implementing an online resource, including an online memorial.

The participants mainly used online resources to obtain information and to reach peers bereaved by suicide. Consistently, in a recent systematic review, Lestienne et al. [13] reported that seeking and sharing support were the most frequent purposes of the use of online resources in people bereaved by suicide. Seeking contact with peers [21], discussing with other bereaved people [19,22], starting new threads of discussion and actively seeking support from other members [19,23] are known to be reasons for joining online resources. At the same time, offering help and support to others by replying to messages [23], enabling other users to offer help to cope with their distress [24] and providing support [19,25] were also reported as reasons to use online resources. Some studies notably raised the importance of the Internet as a safe place to discuss taboo or stigmatised topics [22,23,24].

While the use of online resources was reported as frequent during the bereavement process, the participants also reported that existing online resources are insufficient. Moreover, the satisfaction regarding the use of online resources was low. These results indicate the need to implement high-quality online resources dedicated to people bereaved by suicide. The following steps of our study aim to develop an adaptive and online resource through a mixed-method user-centred design [13]. Only a minority of the sample reported the use of online resources as not beneficial. However, this result indicates that further assessment of the positive and negative effects of future online resources will be critically needed after their implementation [13,20].

Interestingly, receiving counselling was found to be associated with greater expectancies regarding future online resources, meaning that online resources are seen as an addition, rather than a substitute, for other sources of support, such as face-to-face counselling or peer support groups. This was also reported in previous studies [13]. In their survey of 104 parents bereaved by suicide using Internet support groups and 297 parents using face-to-face groups, Feigelman et al. [24], for example, reported similar sociodemographic and clinical characteristics in both groups. More precisely, online and face-to-face resources could be complementary and jointly enhance access to help and support for people bereaved by suicide. For example, the participants in our survey more frequently expected online resources to help them in seeking counselling rather than directly offering online counselling.

Unexpectedly, we found no significant discrepancies between women and men regarding the expectations towards online resources, while women reported a greater use of memorialising. Women are known to be more prone than men to engage in postvention resources [26], but our results tended to report that men display similar expectations to women. However, given the low number of men participants in our study, this absence of difference could be due to a lack of statistical power between the two groups. Younger participants were more prone to expect the online resource to help them in seeking counselling. This result indicates that online resources may constitute an interesting means of improving the engagement of younger people in postvention resources. Thus, online resources must be designed to adequately and equally fill the needs of several sociodemographic categories of people bereaved by suicide, including men, women and youth.

The group of parents bereaved by the suicide of their child also tended to report specific patterns of use and expectations regarding online resources for their grief process. This population is known to be at higher risk than other bereaved people for negative mental health outcomes [27,28]. Thus, online resources should specifically address the needs of parents bereaved by suicide by offering them information on their grief process, facilitating their early access to counselling or to other parents bereaved by suicide in order to share their distress. In their longitudinal qualitative study, Entili et al. [29], for example, reported changing needs and coping strategies over a 24-month period, with differentiation between strategies adopted by fathers and mothers.

Social stigma is known to be highly prevalent in people bereaved by suicide and constitutes a critical barrier to accessing face-to-face support [6,30]. In a survey performed in Israel, Nuttman-Schwartz et al. [31] reported that the general population mostly considered suicide bereavement as a “private” concern that does not involve any duty for the state to provide assistance to suicide survivors. Moreover, secrecy, shame or cultural repression related to suicide and suicide bereavement can negatively impact those bereaved by suicide [2,32]. Interestingly, online resources offer a safe space to open the dialogue on suicide bereavement by providing an interface between people bereaved by suicide, mental health professionals and volunteers. Indeed, the very properties of online social spaces are likely to lower inhibitions, facilitate engagement and ease the motivational cost of seeking help [13]. Moreover, developing an online resource may also improve a sense of legitimacy for those bereaved by suicide, as users can feel free to share their emotions, feelings and thoughts about their loss without any social judgement. Accordingly, several authors [33,34] recently discussed how the Internet is offering new forms of mourning by providing a space to subject the living and the dead through a redefinition of time and space and of private and public realms. Walter et al. [34], for example, argued that online practices have strong implications on key concepts of bereavement, such as the disenfranchisement of grief, private grief, social death or continuing bonds with the deceased. Particularly, online resources can bring dying and grieving out of both the private and public realms beyond the immediate family and provide an audience for once private communications on bereavement. They may, thus, help those bereaved in sharing and communicating emotions and feelings that can remain unexpressed in the private circle, such as guilt, blame or emptiness.

According to our results, the development and implementation of evidence-based online resources dedicated to suicide bereavement are urgently needed. Based on our results and a recent expert consensus on postvention interventions [35], several recommendations can be provided for the development of such resources. First, a web platform available through computers and cell phones appears to be the most expected resource by a vast majority of people bereaved by suicide. Second, the resource must mainly offer reliable and updated information on suicide prevention and on suicide bereavement. Third, access to peers bereaved by suicide should also be available through the online resource. Fourth, the special needs of some categories of people bereaved by suicide (especially parents bereaved by the suicide of a child or youth) must be filled by the resource. The proposed digital resource must, thus, be protean in order to propose affording and singularised resources for everyone. Finally, helping the users of the resource in finding where to receive counselling can reinforce the complementarity between face-to-face and online support.

### 4.3. Strengths and Limitations

We performed the largest online survey on the needs, use and expectations of people bereaved by suicide regarding online resources. With a total of 401 participants, we exceeded the number of 385 participants, which has been shown to offer convenience sample surveys [18]. Our data can help stakeholders to implement online resources dedicated to people bereaved by suicide by offering precise insights into their effective use and expectations about such resources.

However, our study has several limitations. First, the vast majority of participants were women, which may impede the generalizability of our results. However, the ratio is consistent with those of previous studies on suicide bereavement [13,19]. This may be explained by a higher propensity for bereaved women to participate in research compared to bereaved men [13,19,36], but also by the higher prevalence of suicide bereavement in women compared to men, as men are more prone to die by suicide [37]. Second, using an online survey may have induced a selection bias, as people who do not often use online resources may have been less prone to participate in our study. It is notable that the vast majority of the participants reported daily use of the Internet and social media. Third, our sample was recruited in France, which may also have impeded the generalisability of our results. However, the consistency of our results with previous studies on the topic indicates that they could be transposed into other sociocultural contexts. Finally, we did not assess the role of social and emotional responses in the use or expectations regarding online resources. Notably, the roles of secrecy and shame related to suicide or of cultural repression of grieving were not studied through our questionnaire. Further studies should assess how online resources can be effective in reducing the taboo and stigma of suicide and suicide bereavement by helping people bereaved by suicide to freely express and share their feelings and emotions.

## 5. Conclusions

We performed a large national survey on the needs, use and expectations of people bereaved by suicide regarding online resources. The majority of people bereaved by suicide use the Internet for their bereavement process, mainly to obtain information on suicide bereavement and suicide prevention and to access testimonies of other people bereaved by suicide. However, available online resources for people bereaved by suicide are insufficient. Thus, the development of web platforms offering access to reliable information on suicide bereavement and on suicide prevention to peers bereaved by suicide and help to seek counselling are urgently needed.

## Figures and Tables

**Table 1:** Characteristics of the participants.

	N	%
**Age (mean +/− SD, N = 399)**	45.7	(SD = 12.7)
**Gender (female)**	354	88.3
**Status of the deceased**		
Child	133	33.17
Sibling	56	13.97
Partner	59	14.71
Parent	56	13.97
Other	97	24.19
**Duration of bereavement**		
>3 years	201	50.12
<3 years	200	49.88
**Counselling**	254	63.34
Psychotherapy	129	32.17
Psychological counselling	99	24.69
Psychiatric counselling	72	17.96
Group therapy	22	5.49
Associative counselling	71	17.71
**Frequency of Internet use**		
Daily	369	92.48
Weekly	24	6.02
Occasionally	6	1.50
**Frequency of social media use**		
Daily	308	77.58
Weekly	48	12.09
Occasionally	41	10.33
**Available digital devices**		
Computer	298	74.31
Smartphone	379	94.51
Digital tablet	102	25.44

**Table 2:** Reported use of digital devices and online resources during the bereavement process.

	N	%
**Digital devices used**		
Computer	140	34.91
Smartphone	260	64.84
Digital tablet	40	9.98
Other	4	1.00
**Use of the Internet (yes)**	296	73.82
**Perceived benefits**		
Very beneficial	39	11.61
Beneficial	59	17.56
Quite beneficial	81	24.11
Moderately beneficial	74	22.02
Little beneficial	54	16.07
Mostly not beneficial	14	4.17
Not beneficial	15	4.46
**Use of social media (yes)**	247	61.60
**Type of social media**		
Facebook	233	58.10
Twitter	12	2.99
Instagram	33	8.23
Whatsapp	43	10.72
Other	26	6.48
**Perceived benefits**		
Very beneficial	34	11.37
Beneficial	44	14.72
Quite beneficial	81	27.09
Moderately beneficial	62	20.74
Little beneficial	45	15.05
Mostly not beneficial	15	5.02
Not beneficial	18	6.02

**Table 3:** Use and expectations regarding online resources dedicated to suicide bereavement.

	Use of Available Resources			Expectations Regarding a Potential New Online Resource Dedicated to Bereavement by Suicide
	**N**	**%**	**N**	**%**
**Accessing peer support**	231	57.61	263	65.59
**Finding information on suicide**	286	71.32	310	77.31
**Seeking counselling**	15	3.74	235	58.60
**Memorialising**	123	30.67	162	40.40

**Table 4:** Multivariate analysis of factors associated with the use of the Internet.

	Reaching Peers	Obtaining Information	Seeking Counselling	Memorialising
**Age (<46 years)**	1.389 (0.812–2374)	1.356 (0.784–2.345)	1.493 (0.376–5.933)	2.115 (1.154–3.877) *
**Gender (female)**	1.978 (0.987–3.965)	1.784 (0.914–3.483)	-	2.286 (1.003–5.210) *
**Status of the deceased (child)**	8.155 (3.931–16.920) **	2.752 (1.313–5.768) **	2.313 (0.518–10.333)	3.618 (1.696–7.717) **
**Status of the deceased (partner)**	3.245 (1.559–6.757) **	2.043 (0.902–4.627)	-	0.886 (0.398–1.973)
**Status of the deceased (parent)**	1.995 (0.979–4.067)	0.989 (0.482–2.029)	0.760 (0.080–7.230)	0.611 (0.262–1.426)
**Status of the deceased (sibling)**	1.675 (0.821–3.415)	1.035 (0.502–2.133)	1.345 (0.228–7.942)	1.104 (0.507–2.402)
**Duration of bereavement (<3 years)**	1.758 (1.122–2.753) *	1.328 (0.831–2.122)	6.231 (1.367–28.398) *	1.451 (0.926–2.275)
**Counselling (yes)**	1.657 (1.029–2.666) **	2.342 (1.436–3.819) **	6.970 (0.883–55.025)	0.856 (0.517–1.415)

* *p* < 0.05; ** *p* < 0.01. The results are given in odds ratios with 95% confidence intervals. The odds ratios could not be measured for the association between gender/status of the deceased (partner) and seeking counselling due to the low number of responses in some categories of participants to this question.

**Table 5:** Multivariate analysis of factors associated with the expectations regarding online resources.

	Reaching Peers	Obtaining Information	Seeking Counselling	Memorialising
**Age (<46 years)**	1.447 (0.866–2.418)	1.601 (0.901–2.847)	2.020 (1.214–3.360) **	1.569 (0.929–2.651)
**Gender (female)**	1.028 (0.536–1.970)	1.559 (0.777–3.130)	1.486 (0.786–2.809)	1.739 (0.877–3.446)
**Status of the deceased (child)**	2.153 (1.093–4.240) *	1.754 (0.801–3.841)	1.651 (0.842–3.236)	1.354 (0.693–2.645)
**Status of the deceased (partner)**	1.446 (0.705–2.968)	0.630 (0.284–1.397)	0.635 (0.314–1.285)	0.317 (0.146–0.690) **
**Status of the deceased (parent)**	1.623 (0.798–3.301)	1.760 (0.732–4.234)	1.189 (0.586–2.416)	0.481 (0.231–0.998) *
**Status of the deceased (sibling)**	1.634 (0.800–3.338)	0.700 (0.323–1.516)	1.227 (0.602–2.499)	1.170 (0.587–2.329)
**Duration (<3 years)**	0.977 (0.636–1.502)	1.077 (0.653–1.775)	0.789 (0.518–1.201)	1.319 (0.868–2.005)
**Counselling (yes)**	1.696 (1.068–2.695) *	2.913 (1.709–4.965) **	2.156 (1.360–3.418) **	1.168 (0.734–1.861)

* *p* < 0.05; ** *p* < 0.01. The results are given in odds ratios with 95% confidence intervals.

## Data Availability

The data that support the findings of this study are available from the corresponding author, E.L., upon reasonable request.

## Supplementary Materials

The following supporting information can be downloaded at: https://www.mdpi.com/article/10.3390/ijerph191912156/s1.
