# Supplementary material for: The Needs, Use and Expectations of People Bereaved by Suicide Regarding Online Resources: An Online Survey
Source: Int J Environ Res Public Health. 2022 Sep 26;19(19):12156. doi: 10.3390/ijerph191912156 (PMC9566504; doi:10.3390/ijerph191912156)
Supplement: Supplementary file 1 [file ijerph-19-12156-s001.zip › ijerph-1869493-supplementary.pdf]

Supplementary material. Questionnaire used for the online survey

A. Sociodemographic and loss-related characteristics

A.1. What is your gender?

- ☐ Male
- ☐ Female
- ☐ Non-binary

A.2. How old are you? __________

A.3. Who was your relative you died by suicide?

- ☐ Your son
- ☐ Your daughter
- ☐ Your partner
- ☐ Your brother
- ☐ Your sister
- ☐ Your father
- ☐ Your mother
- ☐ Your grandson
- ☐ Your granddaughter
- ☐ Your grandfather
- ☐ Your grandmother
- ☐ Your cousin
- ☐ Your stepfather
- ☐ Your stepmother
- ☐ Your stepson
- ☐ Your stepdaughter
- ☐ Your friend
- ☐ Your colleague
- ☐ Other

A.4. How long ago did your relative die by suicide?

- ☐ Less than 6 months
- ☐ 6 months to 1 year
- ☐ 1 to 3 years
- ☐ 3 to 5 years
- ☐ 5 to 10 years
- ☐ 10 to 20 years
- ☐ More than 20 years

A.5. Did your receive counselling after the death of your relative?

- ☐ Yes
- ☐ No

A.6. If yes, which type(s) of counselling did your received?

- ☐ Individual psychotherapy
- ☐ Psychological counselling
- ☐ Psychiatric counselling
- ☐ Collective psychotherapy
- ☐ Peer support group

B. Use of the Internet and social media in daily life

B.1. How often do you use the Internet?

- ☐ Never
- ☐ Less than once a month

- ☐ Several times a month
- ☐ Several times a week
- ☐ Several times a day
- ☐ Many times a day

B.2. How often do you use social media?

- ☐ Never
- ☐ Less than once a month
- ☐ Several times a month
- ☐ Several times a week
- ☐ Several times a day
- ☐ Many times a day

B.3. What type of digital resource(s) do you have access to?

- ☐ Computer
- ☐ Laptop
- ☐ Smartphone
- ☐ Digital tablet

C. Loss-related needs during suicide bereavement

To what extent did you experience the followings needs after the death of your relative?

	**Not at all**	**A little bit**	**Moderately**	**Quite a bit**	**Extremely**
Accessing testimonies of people bereaved by suicide					
Chatting with people bereaved by suicide					
Being supported by people bereaved by suicide					
Finding information on suicide					
Finding information on suicide bereavement					
Discussing with a mental health professional					
Seeking for counselling					
Receiving online counselling					
Memorializing					
Finding how to talk about suicide with your relatives					
Find inghow to talk about suicide with children					

D. Use of the Internet and social media during suicide bereavement

D.1. In the days and weeks following the death of your relative, did you use the Internet?

- ☐ Yes
- ☐ No

D.2. If yes, you did use the Internet to:

- ☐ Announce the death of your relative
- ☐ Access testimonies of people bereaved by suicide
- ☐ Chat with people bereaved by suicide
- ☐ Be supported by people bereaved by suicide
- ☐ Find information on suicide
- ☐ Find information on suicide bereavement
- ☐ Discuss with a mental health professional
- ☐ Seek for counselling
- ☐ Receive online counselling
- ☐ Memorialize
- ☐ Find how to talk about suicide with your relatives

- ☐ Find how to talk about suicide with children

D.3. If yes, how much did you perceive the use of the Internet as beneficial?

- ☐ Not beneficial
- ☐ A little bit beneficial
- ☐ Moderately beneficial
- ☐ Quite a bit beneficial
- ☐ Beneficial
- ☐ Extremely beneficial

D.4. In the days and weeks following the death of your relative, did you use social media?

- ☐ Yes
- ☐ No

D.5. If yes, which social media did you use?

- ☐ Facebook
- ☐ Twitter
- ☐ Instagram
- ☐ WhatsApp
- ☐ LinkedIn
- ☐ Reddit
- ☐ Snapchat
- ☐ TikTok
- ☐ Flickr
- ☐ Other

D.6. If yes, you did use social media to:

- ☐ Announce the death of your relative
- ☐ Access testimonies of people bereaved by suicide
- ☐ Chat with people bereaved by suicide
- ☐ Be supported by people bereaved by suicide
- ☐ Find information on suicide
- ☐ Find information on suicide bereavement
- ☐ Discuss with a mental health professional
- ☐ Seek for counselling
- ☐ Receive online counselling
- ☐ Memorialize
- ☐ Find how to talk about suicide with your relatives
- ☐ Find how to talk about suicide with children

D.7. If yes, how much did you perceive the use of social media as beneficial?

- ☐ Not beneficial
- ☐ A little bit beneficial
- ☐ Moderately beneficial
- ☐ Quite a bit beneficial
- ☐ Beneficial
- ☐ Extremely beneficial

D.8. Which type(s) of digital resource did you use to go on the Internet or social media?

- ☐ Computer
- ☐ Laptop
- ☐ Smartphone
- ☐ Digital tablet

D.9. Please add any additional comment on your use of the Internet or social media during suicide bereavement: ____

E. Expectations regarding online resources for people bereaved by suicide

E.1. In your opinion, online resources dedicated to suicide bereavement are:

- ☐ Largely insufficient
- ☐ Insufficient
- ☐ Quite sufficient
- ☐ Sufficient
- ☐ Largely sufficient

E.2. In your opinion, which type(s) of online resources would be the most useful for people bereaved by suicide?

- ☐ Website
- ☐ Mobile app
- ☐ Social media
- ☐ Chatbot

E.3. To what extent would you expect that the online resource allows to:

- ☐ Access testimonies of people bereaved by suicide
- ☐ Chat with people bereaved by suicide
- ☐ Be supported by people bereaved by suicide
- ☐ Find information on suicide
- ☐ Find information on suicide bereavement
- ☐ Discuss with a mental health professional
- ☐ Seek for counselling
- ☐ Receive online counselling
- ☐ Memorialize
- ☐ Find how to talk about suicide with your relatives
- ☐ Find how to talk about suicide with children

E.4. Would you find it useful if the users of the resource could chat together?

- ☐ Yes
- ☐ No

E.5. Would you find it useful if the users of the resource could add some content?

- ☐ Yes
- ☐ No

E.6. Would you find it useful if some content was available only for users registered on the resource?

- ☐ Yes
- ☐ No

E.7. Please add any additional comment on your expectations:

____
